# Dried Blood Spot Tests for the Diagnosis and Therapeutic Monitoring of HIV and Viral Hepatitis B and C

**DOI:** 10.3389/fmicb.2020.00373

**Published:** 2020-03-09

**Authors:** Edouard Tuaillon, Dramane Kania, Amandine Pisoni, Karine Bollore, Fabien Taieb, Esther Nina Ontsira Ngoyi, Roxane Schaub, Jean-Christophe Plantier, Alain Makinson, Philippe Van de Perre

**Affiliations:** ^1^Pathogenèse et Contrôle des Infections Chroniques, INSERM U1058, Centre Hospitalier Universitaire de Montpellier, Montpellier, France; ^2^Centre Muraz, Bobo Dioulasso, Burkina Faso; ^3^Emerging Diseases Epidemiology Unit, Center for Translational Science, Institut Pasteur, Paris, France; ^4^CIC AG/INSERM 1424, Centre Hospitalier de Cayenne, Cayenne, France; ^5^Laboratoire de Virologie, Normandie Université, CHU de Rouen, Rouen, France; ^6^INSERM U1175/IRD UMI 233, IRD, CHU de Montpellier, Montpellier, France

**Keywords:** dried blood spot, hepatitis B, hepatitis C, HIV, diagnosis, hard-to-reach population

## Abstract

Blood collected and dried on a paper card – dried blood spot (DBS) – knows a growing interest as a sampling method that can be performed outside care facilities by capillary puncture, and transported in a simple and safe manner by mail. The benefits of this method for blood collection and transport has recently led the World Health Organization to recommend DBS for HIV and hepatitis B and C diagnosis. The clinical utility of DBS sampling to improve diagnostics and care of HIV and hepatitis B and C infection in hard to reach populations, key populations and people living in low-income settings was highlighted. Literature about usefulness of DBS specimens in the therapeutic cascade of care – screening, confirmation, quantification of nucleic acids, and resistance genotyping -, was reviewed. DBS samples are suitable for testing antibodies, antigens, or nucleic acids using most laboratory methods. Good sensibility and specificity have been reported for infant HIV diagnosis and diagnosis of hepatitis B and C. The performance of HIV RNA testing on DBS to identified virological failure on antiretroviral therapy is also high but not optimal because of the dilution of dried blood in the elution buffer, reducing the analytical sensitivity, and because of the contamination by intracellular HIV DNA. Standardized protocols are needed for inter-laboratory comparisons, and manufacturers should pursue regulatory approval for *in vitro* diagnostics using DBS specimens. Despite these limitations, DBS sampling is a clinically relevant tool to improve access to infectious disease diagnosis worldwide.

## Introduction

The use of dried blood collected on blotting paper, – “Dried Blood Spot” (DBS) -, was developed gradually after the Second World War. The beginning of diagnosis on DBS is associated with Robert Guthrie who implemented large-scale neonatal screening for phenylketonuria in the 1960s. In the field of infectious disease diagnosis, the first references to DBS relate to syphilis, with studies published as early as the 1950s (Blaurock et al., 1950). The DBS sample was also used for serological surveillance or diagnosis of trypanosomiasis, hepatic amebiasis, congenital rubella, or hepatitis B (Ashkar and Ochilo, 1972; Ambroise-Thomas and Meyer, 1975; Farzadegan et al., 1978; Sander and Niehaus, 1980). After these modest and historic beginnings, a progressively increasing interest for DBS was observed in the early 2000’s, mainly due to the needs of therapeutic monitoring of HIV infection.

The blood can be directly deposited on the filter paper when a capillary blood collection is done with a retractable incision device, or using a pipette when peripheral venous blood is collected. The procedure for DBS collection has been detailed (Ostler et al., 2014). The capillary sampling on DBS is performed at the heel of the infant and at the level of the digital pulp in children and adults. Self-sampling is possible after a minimum training. For digital samples, the fingers innervated by the ulnar nerve (half of the 3rd finger and 4th or 5th finger) are generally preferred because less sensitive than fingers innervated by the median nerve. The size of the skin penetration depends on the size and type of lancet, and determines the amount of blood that can be collected. Standard DBS cards use pre-printed circles 12 mm in diameter to receive between 50 and 70 μl of blood. The massage of the finger before puncture and warming of the hands in warm water, can facilitate the sampling. After puncture it is important to exert a strong intermittent pressure to maintain the bleeding and complete the blotting paper card.

## Indications of DBS in Infectious Diseases

Dried blood spot sampling offers an alternative to the reference samples – plasma and serum – in situations where there are no facilities or expertise to take venous whole blood specimens; or where transport of body fluids is difficult. Hence, DBS specimens collected outside of healthcare facilities can be viewed as an alternative to rapid diagnostic tests (RDT). Compared to RDT, DBS sampling has advantages and limits summarized in Table 1. By comparison with RDT one of the main limit is that diagnostic approach using DBS request a post-test visit after the sampling which is associated with a risk of loss to follow up. On the other hand, it is useful to test a large number of persons by using DBS testing in a centralized laboratory, and it is possible to confirm the infections with Western blot and molecular tests. Hence, in countries with high economic resources, DBS can be used to promote access to screening in key populations having little use of care facilities. Screening programs using DBS have been running for several years in Great Britain, targeting in particular intravenous drug users. Up to 20% of new HCV diagnoses were made using DBS in 2013 in Scottish addiction treatment centers (McLeod et al., 2014). A program of this type has been implemented by the Montpellier teaching hospital since 2013 in all addiction treatment centers located in the Languedoc Roussillon Region (Accueillir, 2014). Sex workers (Shokoohi et al., 2016), homeless people (Foroughi et al., 2017), migrants and some men who have sex with men (MSM) (Bogowicz et al., 2016), as well as populations living in hard to reach areas such as French Guiana (Schaub, 2017) are also key populations that can have difficult access to laboratory infectious diseases testing for which the use of DBS should be considered.

**TABLE 1:** Comparison of the characteristics of DBS tests and rapid diagnostic tests.

	DBS	Rapid Tests
Capillary blood	Yes	Yes
Hard to reach populations	Yes	Yes
Immediate result (<15 min)	No	Yes
Return visit request	Yes	No
CE-IVD, FDA, WHO prequalification	No*	Yes
Based on laboratory expertise	Yes	No
Useful for all diagnosis steps	Yes	No

*Except for HIV RNA nucleic acid tests. CE-IVD, CE-marked In Vitro Diagnostic Medical Device; FDA, Food and Drug Administration. Advantages of DBS tests are indicated in green and limits in red.

In resource-limited settings the high rates of infectious disease mortality and morbidity are, to a large extent, due to a lack of diagnostic means (Global Health Workforce Alliance and World Health Organization [WHO], 2013). Insufficient access to nearby laboratory facilities is a major concern. The lack of adequate human and financial resources – health professionals and biologists – are also noteworthy. It is estimated that three-quarters of Africa’s population have access to minimal health care structures in Africa, and more than 90% in Asia. By contrast, less than one-third of Africans would have access to advanced health facilities and just over 50% in Asia (RAND Corporation, 2007. Estimating the global health impact of improved diagnostic tools for the developing world; Abou Tayoun et al., 2014).

**FIGURE 1:**
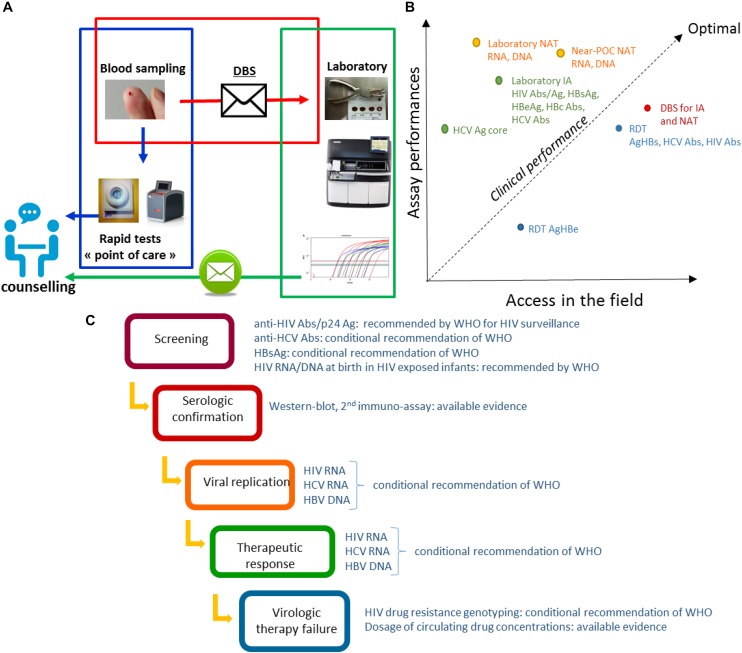
**(A)** Possible organization of the diagnosis and management of infections combining rapid diagnostic tests and DBS. The sampling is carried out closer to the person detected or supported. Non-laboratory RDT are carried out in the peripheral structures in parallel with the sending of the DBS to the central laboratory carrying out complementary or confirmation analyses. Results are reported as part of the post-test counseling. **(B)** Clinical performance of *in vitro* assays dedicated to HBV, HCV, and HIV infections. The figure is a schematic representation of the clinical performances considered as a trade-off between assay performances and implementation coverage. Diagnosis and monitoring strategies based on different formats of tests have different clinical performances. Each format of test is characterized by its analytical performances mainly estimated by lower limit of detection (LOD), sensibility (Se) and specificity (Sp) based on previously published studies, and its global accessibility depending on parameters such as price, infrastructure requirements, distribution network, and acceptability as evaluated based on our own experience. The clinical performance in a population can be estimate by the proportion of infected persons tested and detected positive. Abs, antibodies; Ag, antigen; HIV Abs, anti-HIV antibodies; HCV Abs, anti-HCV antibodies; IA, immuno-assay; NAT, Nucleic acid tests; Near-POC NAT, Near point of care NAT; RDT, rapid diagnostic test. **(C)** DBS analyses during the therapeutic cascade for HIV, HBV, and HCV infections. The sampling on DBS allows the realization of the *in vitro* assays which are necessary at each steps of the therapeutic cascade: screening, confirmation, measurement of the replication, analysis of the therapeutic failures. Recommendations of WHO to the usage of DBS are indicated for each step of the cascade.

Point of care (POC) tests – usable outside healthcare facilities – and technologies requiring minimal infrastructure (near-POC tests), constitute a major progress but will require significant investments to expand the limited range of analyzable parameters. Necessity of machines maintenance, reagents supply and quality control assurance need also to be considered in a medium and long term approach for decentralized laboratories. Good acceptability of capillary blood sampling is another advantage of DBS when compared with venepuncture (Pell et al., 2014; Almond et al., 2016). In this context, sampling on DBS considers decentralized sampling as possible, whilst carrying out the test in a well-equipped centralized clinical laboratory, without waiting for the uncertain availability of new technologies and without necessity of maintaining a cold chain for transportation (Figure 1A). Transportation and storage in field situations can have a significant effect, as shown for HIV and HCV nucleic amplifications (Monleau et al., 2010; Tuaillon et al., 2010; Manak et al., 2018), and HCV antibodies testing (Marques et al., 2012). The filter papers used can also impact on the performances of *in vitro* diagnosis tests. Among the DBS collection cards available Whatman 903, Munktell TNF or Ahlstrom Grade 226 have been recommended but other cards have also demonstrated good performances (Waters et al., 2007; Rottinghaus et al., 2013; Smit et al., 2014; World Health Organization [WHO], 2014; Taieb et al., 2016). DBS specimens should be considered from a public health perspective, for which the clinical performance of the *in vitro* laboratory assays is crucial. The clinical performance of a test can be analyzed as a trade-off between the intrinsic performances of the assay and its accessibility in the field. The best clinical performances are obtained in populations in whom the highest proportion of infected persons are tested and detected positive. High clinical performances may be achieved using DBS based strategies (Figure 1B). Implementation of DBS for HIV viral load is considered as one of the most medically effective immediate measures to reduce AIDS-related mortality in Africa (Phillips et al., 2015).

In addition to individual diagnosis, sampling, transport and storage simplification makes the DBS a particularly suitable tool for population studies. In France the mandatory reporting system for HIV is associated with virological surveillance by the HIV National Reference Center using dried serum spot (Lot et al., 2004). The HIV National Reference Center identifies HIV types, groups, and subtypes, and estimates incidence using a recent infection test. This surveillance system provides robust and comprehensive data on the HIV epidemic in France. Other countries, such as Germany, have also integrated DBS into their HIV surveillance system thanks to the DBS ease (Hofmann et al., 2017). In southern countries and areas with difficult access the DBS allows large-scale surveys to plan and monitor health programs. DBS specimens collected during Demographic and Health Surveys (DHS) are useful for estimating the prevalence of diseases, allowing reliable countrywide and regional distribution of HIV estimates (Bellan et al., 2013), but also of hepatitis B, C, and delta, as recently reported (Meda et al., 2018; Njouom et al., 2018; Tuaillon et al., 2018).

## DBS in the Therapeutic Cascade of Care

Reaching and testing persons at risk of HBV, HCV, and HIV is a main challenge as part of the global effort to eliminate these infections as public health threats by 2030 (World Health Organization [WHO], 2016). Diagnosis of viral hepatitis and HIV follows a sequential strategy initiated by serological screening based on the detection of antibodies or antigens, to which succeeds a confirmation step and the therapeutic monitoring (Figure 1C). DBS analyses can be integrated into each steps of the diagnosis cascade.

### DBS for Screening and Serologic Confirmation of HIV and Viral Hepatitis

Dried blood spot can be used in the initial serological screening step as an alternative to conventional assays based on venipuncture and RDT. DBS can be decentralized such as RDT, but requires a return visit for post-counseling. This second visit leads to a risk of loss to follow up (Bottero et al., 2015). The lower rate of retention in care using DBS is one of the limitations of DBS testing compared to RDT. Nevertheless, questioning health care workers about their perception of DBS testing highlights some advantages related to a diagnostic strategy requiring a second visit: (i) unlike for RDT, the person who performed the DBS does not bear the responsibility of performing the assay and of immediately notifying the diagnosis to the screened subject; (ii) in the event of a positive test, the medical team can anticipate and better organize the post-test counseling; (iii) in an approach of risk-reduction, the time between screening and the result announcement may be perceived as beneficial to raising awareness.

Dried blood spot samples can be used with standard HIV and viral hepatitis immunoassays. Automated immunoassays performed on DBS can be more efficient than RDT based on immuno-concentration, immunochromatography or agglutination methods. We reported that acute HIV infection was detected earlier on DBS using a fourth-generation HIV test (combining detection of total antibody and p24 Ag) compared to RDT (Kania et al., 2015). Suboptimal analytical sensitivity was also reported for HBsAg RDT (Scheiblauer et al., 2010). This analytical sensibility is generally insufficient to meet the minimum requirements of European regulatory authorities and WHO (Lower limit of detection (LOD) < 4 IU/mL) (Chevaliez et al., 2014; World Health Organization [WHO], 2019). HBsAg laboratory tests have a sensitivity <0.1 IU/mL (Tuaillon et al., 2012), suggesting that even with a dilution factor of 10 or 20 times related to the elution step, the DBS testing may have a better sensitivity than HBsAg RDT. Systematic reviews have reported 98% sensitivity and 100% specificity for HBsAg screening on DBS compared to 88.9% sensitivity and 98.4% specificity using HBsAg RDT (Amini et al., 2017; Lange et al., 2017a). By contrast, the overall performances of RDT and DBS test dedicated to anti-HCV detection appeared very close, with 98% sensitivity, and 99% specificity using DBS, and 98% sensitivity and 100% sensitivity using RDT (Lange et al., 2017a; Tang et al., 2017).

The confirmation of a positive screening test is a second and often crucial step in the cascade of care. DBS can be used for confirmation of anti-HIV and anti-HCV detection on Western blot. Results in band patterns are similar to those observed in blood and comparable performances in term of sensitivity and specificity (Tuaillon et al., 2010; Kania et al., 2013; Manak et al., 2018). However, these assays are rarely available in low incomes countries, and were generally expensive. Confirmation by genomic detection on DBS is also an effective means of confirming infections with HCV, HBV, and HIV (Tuaillon et al., 2010; Nhan et al., 2014). Nucleic acid tests on DBS are able to confirm and HIV and HCV replication since the RNA level is generally higher than the LOD in treatment naïve subjects (Smit et al., 2014; Nguyen et al., 2018), and to detect chronic active HBV infections (HBV DNA > 2000 IU/ml). Access to a more efficient diagnostic tool in case of high clinical suspicion despite a negative result of TDR could be considered as another way of DBS use (Kania et al., 2015).

### DBS for HIV and Viral Hepatitis Molecular Testing to Detect Viral Replication, Therapeutic Response and Virologic Theray Failure

One of the most obvious indications of the DBS concerns HIV, HBV, and HCV molecular tests. Testing HIV, HBV, and HCV nucleic acids is necessary for therapeutic initiation or monitoring of the therapeutic response. However, access to nucleic acid tests remains very limited in low income countries. The performances of most commercial HIV-1 RNA assays have been evaluated on DBS: Abbott RealTime, Roche Cobas, Siemens Versant, bioMérieux Nuclisens, Biocentric Generic, Cepheid GeneXpert. The Abbott test has been the most largely used assay, and can be considered as the reference for HIV viral load on DBS. The authors conclude quite consensually on the satisfactory performance of viral load on DBS (Smit et al., 2014). Nevertheless, the variability of the results must be underlined. Sensitivities ranged from less than 80% to almost 100% at the threshold of 1000 HIV RNA/mL recommended by WHO for virological failure, and specificity from less than 60% to nearly 95% for a given test (Smit et al., 2014; World Health Organization [WHO], 2014; Taieb et al., 2016). The viral reservoir of HIV DNA induces a quantification bias when whole blood is used. The HIV DNA contained in infected cells is released into the DBS eluate. We have shown that the risk of over-quantification becomes greater over 10^6^ HIV DNA copies per million of peripheral blood mononuclear cells (Zida et al., 2016). The use of a specific extraction of RNA or DNAse significantly improves the specificity (Rollins et al., 2002). At a threshold of 5000 HIV RNA copies/mL, the techniques on DBS show better performances, but this high threshold implies a deterioration of the negative predictive value for a test of therapeutic failure (World Health Organization [WHO], 2014).

The detection of nucleic acids on DBS also finds a strong clinical indication in the diagnosis of infections transmitted from mother to child. Regarding HIV diagnosis, the presence of maternal antibodies in infants makes serological tests ineffective until at least 1 year of age. Molecular diagnosis of HIV infection on DBS has been shown to be effective (Rollins et al., 2002) and is routinely used in clinical practice for this indication. DBS sampling is finally useful for specialized tests such as HIV resistance genotyping, and antiretroviral dosages (D’Avolio et al., 2010; Monleau et al., 2014). It should be noted that although the clinical relevance of HIV resistance genotyping on DBS is indisputable, it nevertheless faces the difficulty of molecular genotyping on DBS, at the threshold defining the therapeutic failure (Monleau et al., 2014).

For viral hepatitis, there are fewer data on molecular tests, but they show better clinical performance than those published for HIV. For HBV, sensitivities and specificities are estimated at 95% on average (D’Avolio et al., 2010), and the threshold for inactive hepatitis is easily reached in DBS (<2000 IU/mL, approximately equivalent to <10,000 DNA copies/mL). For HCV, mean sensitivities and specificities are also high and estimated >98% (Lange et al., 2017b), which can be explained by the paucity of low HCV viremia during the natural history of this infection. Confirmation of HCV viremia by testing HCV core antigen (HCVcAg) on DBS has also recently been reported in intravenous drug users living in Africa and Asia (Mohamed et al., 2017; Nguyen et al., 2018). This method appeared highly specific and may benefit from substantial stability under prolonged storage conditions but with a lower analytical sensitivity compared to DBS HCV RNA testing (Soulier et al., 2016; Nguyen et al., 2018).

## Serological and Molecular Testing Using DBS in the Field

Heterogeneity in the pre-analytical procedures used for DBS specimens between laboratories influence the result and make comparisons difficult. The size of the spot, the nature of the elution buffer, the elution volume – and therefore the dilution factor -, the extraction technique, impact the performance of the tests. WHO guidelines for the implementation of HIV viral load and viral hepatitis tests stress the need for the manufacturers to provide application notes for the use of DBS, and at best to pursue regulatory approval for *in vitro* diagnostics using DBS specimens (World Health Organization [WHO], 2014, 2017). The bioMerieux Nuclisens and Abbott RealTime HIV-1 viral load kits have obtained regulatory approval for use on DBS. On this matrix, the detection threshold (95% detection) is 802 copies/ml for the bioMerieux test (CE-IVD) and 839 copies/ml for the Abbott test (CE-IVD and WHO prequalification). However, a study has shown that the detection rate does not reach 95% for viral loads between 1000 and 5000 RNA copies/mL (Viljoen et al., 2010). WHO pre-qualification of the Abbott HIV test on DBS, report a sensitivity of 76.0% and a specificity of 89.7% at the threshold of 1000 RNA copies/mL (World Health Organization [WHO], 2018). Several qualitative RNA/DNA tests have regulatory approval for the perinatal diagnosis of HIV infection on DBS: the Cepheid XPERT HIV-1 QUAL test that detects HIV-1 DNA and RNA, the Abbott RealTime Qualitative test, as well as the Roche COBAS qualitative test.

For viral hepatitis B and C, no viral load test performed from DBS is currently IVD approved. The same scarcity situation must be noted for viral serology techniques, for which there is neither regulatory approval for use on DBS nor official application note from manufacturers. Despite these important limitations, DBS collection is recommended by WHO for diagnosis of HIV and viral hepatitis B and C in field settings to improve access to screening and management of populations with poor access to serum tests (Soulier et al., 2016; Taieb et al., 2018).

## Conclusion

Capillary blood collected on blotting paper is an alternative method of sampling with many advantages compared to serum or plasma specimens. The lower analytical sensitivity of assays performed on DBS compared to serum/plasma is one of the limits of DBS, since biomarkers can be present at very low concentration during infection. However, data suggest that the analytical sensitivity of DBS is generally higher than RDT. Another limitation is the lack of commitment by manufacturers to use serological and molecular tests on DBS specimens. The pre-analytical steps of laboratory analyses performed on DBS keep a manual character, including the following steps: to punch out of the spot from each blood-soaked circle, to transfer to the elution tube or plate, to put the tube on a laboratory shaker and let the punched DBSs gently elute for a minimum of 2 h, to transfer the tubes to microcentrifuge to eliminate debris from the supernatants. Diagnostic tests on DBS are consequently difficult to integrate into the laboratory workflow. Hence, the tests on DBS require rigorous validation in clinical laboratories to guarantee the quality of the results. Despite these limitations, DBS is a clinically relevant tool for decentralized sampling. DBS can contribute more broadly to improve access to *in vitro* diagnosis in order to reach the treatment target to help end the AIDS epidemic and to eliminate viral hepatitis as a public health threat by 2030.

## Author Contributions

ET, DK, AP, KB, and PV contributed to the conception and wrote the manuscript. FT, EO, RS, J-CP, and AM participated to wrote and analysis. All authors read and approved the final manuscript.

## Conflict of Interest

The authors declare that the research was conducted in the absence of any commercial or financial relationships that could be construed as a potential conflict of interest.
